# Systematic literature review on technological transformation in SMEs: a transformation encompassing technology assimilation and business model innovation

**DOI:** 10.1007/s11301-023-00327-7

**Published:** 2023-02-24

**Authors:** Camila Silva de Mattos, Giustina Pellegrini, Geoffrey Hagelaar, Wilfred Dolfsma

**Affiliations:** Business Management and Organisation Group, Department of Social Science, Wageningen University and Research, Hollandseweg 1, 6706KN Wageningen, The Netherlands

**Keywords:** Technological transformation, Technology assimilation, Business model innovation, SMEs, Technological innovation, Systematic literature review, M10

## Abstract

Small and medium-sized enterprises (SMEs) are pushed to introduce new technologies due to different requirements and changes in the business setting. The SMEs' transformation to exploit new technologies is challenging given their lack of resources and the complexity of technological transformation, which encompasses technology assimilation and business model innovation (BMI). Although studies recognize the complementarity of technology assimilation and BMI for benefiting from technology, the literature is fragmented, and the technological transformation phenomenon remains abstract, especially in the SMEs' context. To improve understanding of technological transformation in SMEs, a systematic literature review was performed on 165 peer-reviewed papers published from 1999 to 2022, building upon BMI and technology assimilation constructs. The descriptive analysis outlines the field's evolution in terms of research and technological trends. The content analysis shows that: most papers focus on factors; the literature falls short of providing theoretical conceptualization and guidelines for the technological transformation process; only a few studies are dedicated to assessing the outcomes of technological transformation in SMEs; and the papers suggest that SMEs present a low transformation level. Finally, we inductively built a framework for technological transformation and suggest five research avenues.

## Introduction

The current business setting characterized by environmental changes, new requirements, technological development, and new opportunities pushes SMEs to rethink their traditional production methods and introduce new technologies to remain competitive over time (Paoloni et al. 2022; Priyono et al. 2020). However, introducing a new technology is challenging for SMEs with limited access to external knowledge, unclear innovation strategies (Müller et al. 2021), and scarce time and resources for innovation (Bouwman et al. 2019). Indeed, studies have shown that SMEs struggle to exploit and benefit from new technologies (O’Toole 2003; Nguyen et al. 2015).

Studies highlight the need for technology assimilation^1^ (Baird et al. 2017) and BMI (Alojairi et al. 2019) for SMEs to benefit from new technology. Technology assimilation is needed for efficient technology use (Baird et al. 2017), and BMI is required to exploit the opportunities offered by the technology (Alojairi et al. 2019), as technology per se is not enough to provide value for companies.

In fact, technology assimilation and BMI are not independent of each other, but they interact when introducing new technology, and their interconnectedness is crucial for performance (Smajlović et al. 2019). However, although the complementarity between technology assimilation and BMI for exploiting new technology is acknowledged by previous studies (Chesbrough 2007; Baden-Fuller and Haefliger 2013), little attention has been devoted to connecting these two concepts (Smajlović et al. 2019), especially in the context of introducing technology (Sabatini et al. 2022). To conceptually express the organization's transformation due to the introduction of technology (including technology assimilation and BMI), we denominate it as technological transformation.^2^

Technological transformation as a research field is fragmented and lacks explicit academic attention, especially in the SMEs' context: existing literature reviews focus on the separate streams of technology assimilation (e.g. Ahmad and Siraj 2018) or BMI (e.g. Hidayat et al. 2020). Moreover, studies within this context focus on aspects of BMI or technology assimilation, such as factors or business model (BM) configurations (e.g. Jutla 1999; Groot et al. 2019); or case studies on specific technologies (e.g. Alexander 2003; Priyono et al. 2020), which limits the generalizability of their results. Even the individual streams of BMI (Pucihar et al. 2019; Gatautis et al. 2019) and technology assimilation (Ahmad and Siraj 2018) are not well explored in the SMEs' context.

In recent years, there has been growing scholarly attention on digital transformation (i.e. technological transformation with digital technologies), with recent literature reviews contributing to overall technological transformation literature (e.g. Nadkarni and Prügl 2021; Vial 2021). However, although digital transformation studies contribute to the overall body of knowledge on technological transformation, their insights may not be extended to other technological contexts. Moreover, most digital transformation studies overlook SMEs' context (Petzolt et al. 2022).

In fact, technological transformation in SMEs is a research endeavour spread across different research fields and contexts. The literature fragmentation represents a limit for the current scientific literature on technological transformation in SMEs. While SMEs face pressure to introduce different technologies to comply with multiple requirements, this process is encompassing and risky, and existing studies fail to provide its overview. To the best of the authors' knowledge, there is no literature review aiming to improve the general understanding of technological transformation by explicitly combining technology assimilation and BMI in SMEs. Given the SMEs' relevance in terms of employment and the number of firms worldwide (Cantele et al. 2020), research regarding technology transformation in SMEs is needed.

To further establish the technological transformation field, we conducted a systematic literature review (SLR), including 165 peer-reviewed papers. More specifically, the SLR research aims to provide the state-of-the-art of the research field of technological transformation in SMEs, building upon technology assimilation and BMI insights. The SLR draws on descriptive and content analysis. The descriptive analysis presents the development of the study field (from 1999 to 2022). The content analysis inductively assesses the current stage of literature on technological transformation by explicitly merging BMI and technology assimilation concepts. Moreover, we classify the papers' foci into research streams and identify drivers, barriers, enablers, control variables, processes, and outcomes for technological transformation. Finally, we derive a framework for technological transformation based on content analysis.

This work contributes to advancing the understanding of the technological transformation phenomenon in SMEs by shedding light on the existing contributions and future research avenues in the field. As technological transformation is a research endeavour dispersed across different research fields, we contribute to the literature as a first step to make it explicit as a concept and to derive insights into the interactive merger between technology assimilation and BMI. This paper is structured as follows. Section 2 presents the research design of the SLR. Section 3 shows the results of the SLR based on descriptive and content analysis. Section 4 shows the discussion of the findings. Finally, Sects. 5 and 6 present the perspectives for future studies and conclusions.

## Research design

This study adopted a systematic literature review (SLR) as a research approach to ensure a transparent, structured, and replicable process, reducing bias and enhancing the data analysis's legitimacy (Tranfield et al. 2003). This work followed the SLR phases suggested by Tranfield et al. (2003): (i) review planning, (ii) conducting the review, and (iii) reporting and dissemination.

In the phase of review planning, a literature analysis on technological transformation in SMEs was conducted. This preliminary investigation showed that BMI and technology assimilation are relevant for effective technological transformation, but these concepts are generally separated in literature (i.e. they are not explicitly connected). We identified two literature reviews (Ahmad and Siraj 2018; Hidayat et al. 2020) focused on factors for technology assimilation and BMI, respectively. Other studies focus on specific aspects of BMI or technology assimilation, such as factors and technology adoption level (e.g. Moeuf et al. 2018). Thus, we identified the need for the SLR because the literature does not provide an overview of technological transformation in SMEs.

In the second phase, the databases Scopus and Web of Science (WOS) were selected because they are the most comprehensive multidisciplinary bibliographic databases (Wang and Waltman 2016). Moreover, using these databases ensures that any paper retrieved would satisfy four of the quality measure's criteria, i.e. CiteScore, source normalized impact per paper, h-index and SCImago journal rank (Nosratabadi et al. 2019). For the articles' search, the keywords related to SMEs, technology, and BMI presented in Table 1 were combined using the Boolean operator “AND” since the objective is to understand technological transformation in SMEs comprehensively. The papers' search was limited to the English language and peer-review journals since these journals can be considered validated knowledge with the highest impact in the field (Crossan and Apaydin 2010).

**Table 1:** Keyword combination

Terms description	Keywords identified for the term
**SMEs:** According to European Commission (2003), SMEs include three categories of enterprises: (i) micro-enterprises, (ii) small enterprises, and (iii) medium-sized enterprises. The search combined all the terms for these categories using the Boolean operator OR	**SMEs:** “SMEs” OR “SME” OR “small and medium enterprises” OR “small and medium-sized enterprises” OR “small and medium businesses” OR “small and medium-sized businesses” OR “small and medium companies” OR “small and medium-sized companies” OR “micro small and medium enterprises” OR “micro small and medium-sized enterprises” OR “MSME” **Micro-enterprises:** “micro enterpris*” OR “micro-sized enterpris*” OR “micro compan*” OR “micro-sized compan*” OR “micro business*” OR “micro-sized business*” OR “micro firm*” OR “micro-sized firm*” **Small enterprises**: “small enterpris*” OR “small-sized enterpris*” OR “small compan*” OR “small-sized compan*” OR “small business*” OR “small-sized business*” OR “small firm*” OR “small-sized firm*” **Medium-sized enterprises:** “medium enterpris*” OR “medium-sized enterpris*” OR “medium compan*” OR “medium-sized compan*” OR “medium business*” OR “medium-sized business*” OR “medium firm*” OR “medium-sized firm*”
**BMI:** The term “business model*” was considered to cover the variety of existing words in the field (e.g. business model, business modelling and business model innovation) and terms used by other literature reviews (e.g. Foss and Saebi 2017)	“Business model*”
**Technology:** We included all terms related to technology introduction/use	“Technolog*” AND (“assimilation” OR “use” OR “implementation” OR “incorporation” OR “integration” OR “infusion” OR “diffusion” OR “adoption” OR “absorption” OR “usage” OR “routinization” OR “routinisation” OR “innovation” OR “transformation” OR “shift*” OR “change*” OR “introduction”)

The articles' selection considered the following filters: (i) elimination of duplicates; (ii) reading of title, abstract, and keywords; and (iii) reading of the full text. The use of EndNote® software facilitated the organization of the retrieved papers. The following criteria were used for screening the articles:

- We discarded papers that do not focus on SMEs' context;
- We are interested in understanding SMEs transforming due to technology introduction/use. Thus, we eliminated papers that present (i) SMEs as technology providers and not as users; (ii) BMI not related to technology; (iii) mention technology-related terms or technology commercialization without addressing technology introduction/use;
- We want to provide an overview of technological transformation by connecting technology assimilation and BMI; thus, we discarded papers that do not address both concepts.

**Fig. 1:**
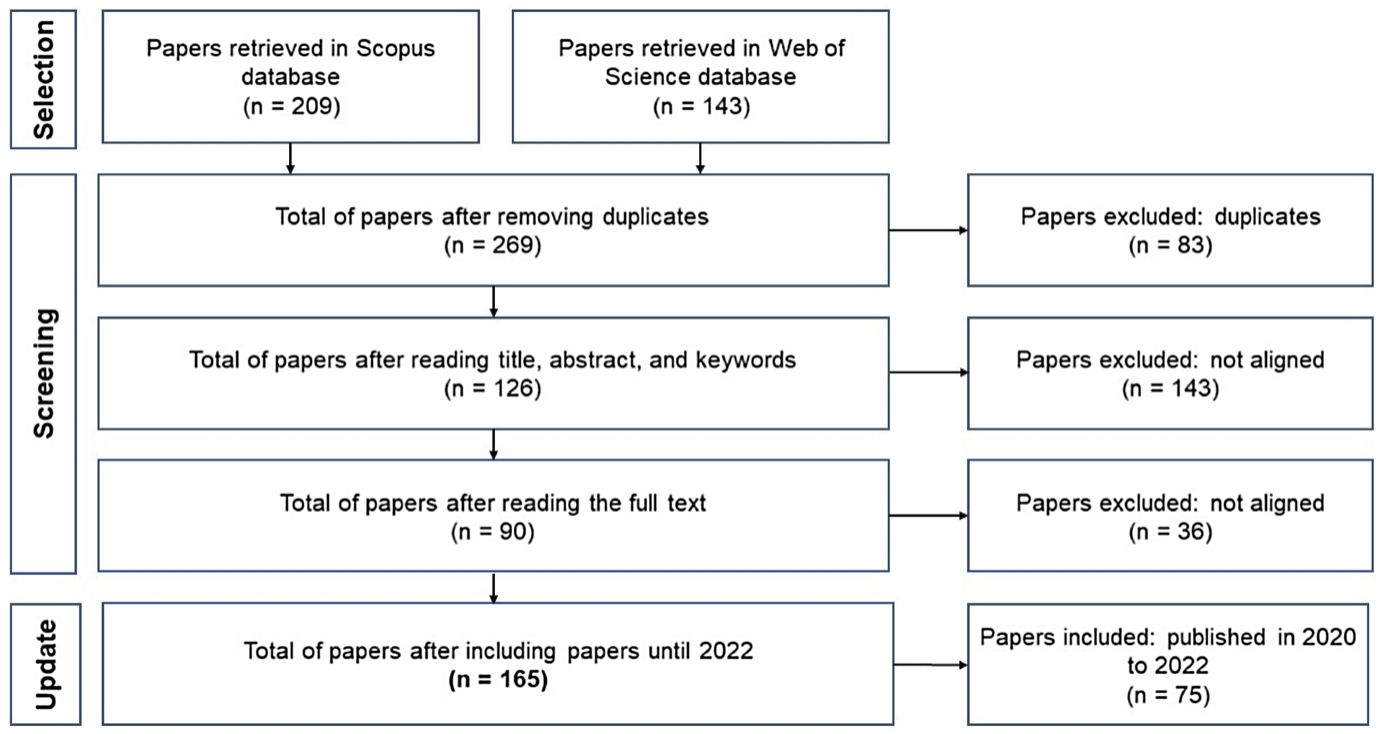
Summary of article selection

The search for papers was performed in November 2020, and the databases recovered 352 papers (209 from Scopus and 143 from WOS), of which 269 were not duplicates. Using the screening criteria, we read the papers' title, abstract, and keywords (resulting in 126 articles), followed by reading the full text (resulting in 90 articles). After that, an update was performed (in December of 2022), using the exact keywords and screening criteria limited to 2020 to 2022 years to update until 2022 (resulting in 75 new papers). The selection resulted in 165 articles (Fig. 1).

The data analysis was conducted through descriptive and content analysis, as Crossan and Apaydin (2010) suggested. We considered categories suggested by Taticchi et al. (2015) for descriptive analysis and included other categories related to our objectives (Table 2).

**Table 2:** Categories—descriptive analysis (Based on Taticchi et al. 2015)

Category	Object of analysis
Number of publications	Size of the research field
Time distribution of publications	Trends in the research field
Technology type	Technological trends in the research field
Popular journals	Main journals where the research is published
Prolific authors	Leading researchers in the field
Local	Setting of the studies
Research approach	Type of studies conducted

The content analysis categories (Table 3) were defined inductively, i.e. after reading and analyzing the retrieved articles (Mayring 2021).

**Table 3:** Categories—content analysis

Category description	References	Note	Section
**Conceptualization:** Conceptualization of technological transformation and classification of papers' foci	Foss and Saebi (2017); Smajlović et al. (2019)	Similarly to Foss and Saebi (2017), we classify the papers' foci based on technology assimilation and BMI dimensions (process or outcome)	3.2.1
**Factors:** Identification and classification of drivers, barriers, and enablers. Drivers positively influence the decision for innovation. Enablers facilitate the innovation process. Barriers negatively affect innovation decision and processes	Groot et al. (2019); Balocco et al. (2012); Hidayat et al. (2020)	The factors identified were organized using the technology–organization–environment (TOE) framework	3.2.2, 3.2.3, 3.2.4
**Process and outcomes**: The process (and the interrelatedness) of technology assimilation and BMI and their outcomes are presented	Foss and Saebi (2017); Ahmad and Siraj (2018); Smajlović et al. (2019)	Based on the work of Smajlović et al. (2019), we infer the relationships between technology assimilation and BMI	3.2.6, 3.2.7, 3.2.8
**Control variables:** The use of controls is common in technology studies, especially when explanatory variables cannot explain data variation	Cruz-Jesus et al. (2019)	–	3.2.5
**Framework:** We organize the factors, processes, control variables and outcomes into a framework	–	–	4

Firstly, we classify the papers' foci by connecting the streams of technology assimilation and BMI. Secondly, we identify and classify the influencing factors for technological transformation. These factors are divided into drivers, barriers, and enablers, consonant with BMI and technology assimilation literature. TOE framework was selected for organizing the factors because it is well established in technology assimilation literature (e.g. Hussein et al. 2019; Ahmad and Siraj 2018) and contains analogous dimensions to internal and external dimensions commonly used in BMI literature (e.g. Foss and Saebi 2017; Pucihar et al. 2019). For classifying the factor as related to technology assimilation and/or BMI, we used the following criteria: (i) we classify the factor according to the classification used by the paper; (ii) if the study does provide a classification, we do it according to the literature on the streams (Foss and Saebi 2017; Ahmad and Siraj 2018). Finally, we inductively build a framework for technological transformation. The results are reported in the next section.

## Results

### Descriptive analysis

Regarding the research approach of the papers, most publications are empirical (90%), and the remainder is theoretical. Most empirical studies are qualitative (56%), followed by quantitative studies (41%) and mixed methods (3%). Section 3.2.1 of the content analysis details the studies' foci.

Regarding the technology type, most of the papers refer to digital technologies (31%), e-commerce (14%), and industry 4.0 (12%). The publications suggest technological trends over time; for instance, there is a concentration of recent papers on digitalization (45% of the articles published from 2019 to 2022) and industry 4.0 (14% of the papers published from 2018 to 2022). These technological trends align with the fourth industrial revolution that demands SMEs to adopt these technologies. Moreover, the role of digital technologies was emphasized during the COVID-19 crisis since they enabled SMEs to keep their operation running with the new restrictions (Akpan et al. 2021). Another technological trend was identified in the first decade of the 2000s: 75% of the papers published from 2002 to 2009 were focused on e-commerce and e-business.

Regarding the time distribution, most of the 165 papers were published in the last 5 years (2017–2022) (around 80%). Until 2014, the number of publications was not higher than three publications per year. Some possible explanations for the recentness of the papers are: BMI is a relatively recent literature outgrowth (Foss and Saebi 2017), and studies on technology assimilation and BMI were previously focused on larger enterprises (Popovič et al. 2014; Pucihar et al. 2019).

We found two journals with more than three publications, namely: Sustainability (fifteen publications) and Technological Forecasting and Social Change (five publications). The prevalence of papers in the Sustainability journal suggests the trend of studies discussing how technologies can generate sustainable opportunities and benefits for companies.

Given the multidisciplinary of the field, we found journals with different scopes with three publications, namely: Journal of Open Innovation: Technology, Market, and Complexity, Industrial Marketing Management, Information Systems Frontiers, Journal of Business Research, Journal of Manufacturing Technology Management, and Journal of Small Business and Entrepreneurship.

The most prolific authors are Julian Müller and Kai-Ingo Voigt (four publications), and their research concerns the 4.0 industry. Two other research clusters were identified: Harry Bouwman, Shahrokh Nikou, and Mark de Reuver, that have two publications in common, and their research is part of the European project “Envision”, which aims to support SMEs in BMI, and Anis Rozmi, Puteri Nohuddin, Abdul Hadi, Mohd Bakar that have two publications in common regarding ICT technologies. The papers were mainly conducted in Europe (52%), followed by Asia (33%), America (11%), Africa (3%), and Oceania (1%), especially in developed countries.

### Content analysis

#### Conceptualization and classification of the papers against the background of technological transformation

Technology assimilation can be defined based on management or system perspectives (Ahmad and Siraj 2018). According to the management perspective, technology assimilation can be defined as an organizational process in which individuals in an organization have their first contact with a technological innovation, which can lead to the adoption, acceptance, utilization, and institutionalization of the innovation (Ahmad and Siraj 2018). For example, Zhu et al. (2006) suggest the three phases that an organization follows to assimilate e-business: (i) e-business initiation, (ii) e-business adoption, and (iii) e-business routinization.

According to the system perspective, technology assimilation can be defined as the extent to which technology use diffuses across organizational projects/work processes and becomes part of their activities (Purvis et al. 2001). For instance, Brown and Lockett (2004) present a five-stage “adoption ladder” for e-business, with each stage representing increased complexity: (i) e-mail, (ii) website, (iii) e-commerce (trading online), (iv) e-business (integrated supply chain), and (v) transformed organization (new BMs based on interworking between organizations). In other words, technology assimilation can be discussed as a process or outcome (level of technology assimilation).

Similarly, BMI can be defined as a process (e.g. experimentation, transformation, search) or as an outcome (innovative BM) in which changes are made in a BM's component (e.g. value proposition, customer segments) or the BM's architecture (Foss and Saebi 2017). BMs are commonly regarded as a representation of the organization's business logic, describing how it creates, delivers, and captures value (Teece 2010). BMI is less well understood than BMs in literature, and although BMI is increasingly gaining attention, it is still undeveloped, especially in the SMEs' context (Ibarra et al. 2020).

BMI and technology assimilation are interconnected (Smajlović et al. 2019), but the studies generally are not integrative. The papers selected do not comprehensively address technological transformation or present an integrative theoretical background to explain it. Generally, the papers that present a theoretical framework rely on BMI theories and/or technology-related theories (e.g. TOE framework, technology acceptance model, etc.). The studies suggest a link between technology assimilation and BMI, but this connection is indirectly inferred. This observation is in line with one of the most cited papers on the nexus between BMI and technology (Baden-Fuller and Haefliger 2013) that state that although they are essentially linked with each other, they are separate constructs.

We classify the papers retrieved based on the recurring subjects, the dimensions of technology assimilation and BMI, and the studies' methods, resulting in identifying six main foci^3^ (Table 4).

1. *Description of innovative BMs for certain technologies:* Papers present new BMs enabled by technologies, which are used as the basis to discuss the new business logic and related processes. The papers' line of reasoning varies according to the nature and recentness of the technologies analyzed. Studies can present criteria to show how certain technologies (and their related BMs) are adequate for SMEs (e.g. Jutla et al. 1999; Laplume et al. 2016). For instance, based on the BM, Laplume et al. (2016) estimate the costs and return on investment (ROI) of additive manufacturing. Some papers present new BMs for new technologies in a particular industry (e.g. Prause 2015, 2016; Leelasantitham 2020). For example, Prause (2016) discusses how e-services for industry 4.0 might look and the complementarity of industry 4.0 and e-residency concepts considering the needs of internationally operating SMEs. Some papers present new opportunities enabled by technologies uncovered by BMs. For instance, Holzmann et al. (2017) present business opportunities (and their related BMs) in 3D printing and classify them according to their attractiveness (costs and potential consumers). Thus, BMs can support decision-making on adopting new technology since it can provide insight into what is feasible for SMEs.
2. *Process of BMI as a consequence of technology assimilation:* The papers describe case studies in which SMEs assimilated new technologies and changed their BM accordingly (e.g. Alexander 2003; Bourdon and Jaouen 2016). Some studies provide details in terms of technology assimilation and BMI. For example, Alexander (2003) describes the transition of a small business in the tourism industry for e-commerce, including the technology assimilation actions (e.g. acquisition of new equipment, provider support, personnel changes) and BMI actions (e.g. changes in value proposition, customer relationships and activities). Other papers describe the BMI side of technological transformation (e.g. Liao 2004; Arnaiz et al. 2016; Sahebalzamani et al. 2022). Few papers provide transformation paths (i.e. how SMEs approach the technological transformation process and the strategies used to cope with its barriers), such as Priyono et al. (2020), that present three digital transformation paths for SMEs. More recent papers address aspects related to decision-making and planning of the technological transformation process. Rozmi et al. (2021) and Azevedo and Almeida (2021) translate the requirements and implications of the transformation process into training programs for decision-makers. Trstenjak et al. (2022) and Petzolt et al. (2022) assess SMEs' readiness and maturity levels.
3. *Technology assimilation implications in BMI:* The studies describe the changes in BMs as a consequence of technology assimilation. These changes can be in BM elements, such as (i) value elements: value creation, value capture, and value offer; or (ii) building blocks: key resources, channels, customer segments, key partners, key activities, customer relations, revenue streams, cost structure, value proposition (e.g. Müller et al. 2018; Norris 2020). Alternatively, changes can be in terms of resulting BMs, such as efficiency-centred and novelty-centred BMs (e.g. Loon and Chik 2019) or a combination of elements of both (Thomson 2022). Studies also analyze the extent to which technologies affected the SMEs' BMs (e.g. Moeuf et al. 2018). In 2022, there is a trend of papers describing the COVID-19 crisis as a precursor of technology adoption and how this context has impacted SMEs' BMs (e.g. Musa et al. 2022; Ludin et al. 2022; Khurana et al. 2022). Finally, one paper predicts the expected changes (through scenarios) with industry 4.0 (Bootz et al. 2022).
4. *Factors that influence technology assimilation and/or BMI:* This is the most popular topic covered by the papers. Papers with this focus describe factors that influence BMI (e.g. Ibarra et al. 2020) and/or technology assimilation (e.g. Mohapatra and Takurta 2019) in the context of technological transformation. The papers describe barriers, drivers, enablers, managerial approaches, and capabilities for technology assimilation and/or BMI (e.g. Groot et al. 2019).
5. *BMI as a precursor of technology assimilation:* The studies discuss BMI as a way to enable SMEs to acquire/use technologies that were not previously feasible. This discussion can take the perspective of (i) technology providers or (ii) users. In the first case, technology providers promote changes in their BMs to offer technology feasibly by changing their value capture (e.g. pay-per-use, license, contracts). For example, Olson et al. (2018) discuss how technology providers offer new ways of delivering ERP software for SMEs. In the second case, SMEs can share resources/installations or engage in ecosystems/collaborative arrangements facilitated by a trusted party (e.g. Brown and Locket 2004; Yang et al. 2019).
6. *Technology assimilation that leads to performance mediated by BMI:* Papers examine whether technology assimilation or technology-driven BMI leads to superior performance. For example, Chen and Zhang (2015) study how e-commerce influences sales growth. Pucihar et al. (2019) investigated performance outcomes of BMI (including technology-driven BMI) in SMEs. The studies within this category are mainly surveys, except for some case studies. For example, Pfister and Lehmann (2022) conducted a qualitative study examining how SMEs use digital technologies to add value and generate ROI. This category is relatively recent within the papers analysed: most studies were published between 2021 and 2022 (80%).

**Table 4:** Research foci of the papers selected

Research focus	Combination of streams	Method	Examples
1. Description of innovative BMs for certain technologies (12%)	Technology assimilation:Technology per se (technology attributes); and BMI: outcome (configuration)	Conceptual Case studies Qualitative studies	Jutla et al. (1999); Balocco et al. (2012); Holzmann et al. (2017)
2. Process of BMI as a consequence of technology assimilation (13%)	Technology assimilation: Process or technology per se; and BMI: process (actions and changes)	Case studies Qualitative studies	Alexander (2003); Apostolov and Coco (2021); Priyono et al. (2020)
3. Technology assimilation implications in BMI (18%)	Technology assimilation: process or outcome; and BMI: outcome (level of BMI)	Case studies Survey data Qualitative studies Conceptual	Müller et al. (2018); Norris (2020)
4. Factors for technology assimilation and/or BMI (37%)	Technology assimilation: process; and/or BMI: process	Case studies Survey data Qualitative studies	Mujahed et al. (2022)**;** Birkel (2019); Rozmi et al. (2020)
5. BMI as a precursor of technology assimilation (7%)	Technology assimilation: process or technology per se; BMI: outcome (configuration)	Case studies Conceptual Qualitative studies	Chan and Chung (2002); Brown and Locket (2004)
6. Technology assimilation that leads to performance mediated by BMI (13%)	Technology assimilation: outcome (level of technology assimilation); BMI: implications in performance	Survey data Case studies	Bouwman et al. (2018); Pucihar et al. (2019)

#### Drivers of technological transformation

The decision to engage in technological transformation can be driven by organizational, environmental, or technological factors or their combination (Müller et al. 2018; Rozmi et al. 2020; Gatautis et al. 2019). Table 5 presents the drivers identified in the papers analyzed and their classification—if they relate to technological assimilation (referred to as TA), BMI, or both.

**Table 5:** Drivers of technological transformation

Technological drivers	Description	References	Classification	
			TA	BMI
Relative advantage	The advantage expected of adopting technology in an organization compared to not adopting it [1]	[1–15]	X	
Perceived usefulness	The degree to which owners/managers believe that employing a technology would improve organization performance [1]	[1, 4, 8, 12, 16–20]	X	
Ease of use	The degree to which an individual believes technology use would be free of effort [18]	[5, 8, 16, 18–21]	X	
Compatibility	The extent to which a technology is appropriate with technology infrastructure, culture, values, and work practices [22]	[5, 17, 20]	X	
Technology readiness	Technology readiness comprises the organization’s technology infrastructure, compatible systems, and employee technical skills [22]	[4, 5, 12, 23]	X	

Environmental drivers	Description	References	Classification	
			TA	BMI
Support of a trusted party	The support of a trusted party provides confidence for SMEs to engage in technological transformation	[2–4, 7, 10, 12, 24–27]	X	X
Existence of feasible BM for SMEs	SMEs have limitations that need to be considered to make it feasible for them to adopt new technologies	[6, 7, 26–30]		X
Competitive pressure	Moves of competitors influence the decision on innovation activities [3]	[2–4, 12, 14, 17, 31–33]	X	X
Customer and/or supplier pressure	Customer and/or supplier request some standards of SMEs related to the use of technologies (e.g. standardized data exchanged) [**3**]	[2–5, 9, 17, 25, 33–35]	X	X
Government regulations	Policy regulations drive the adoption of new technology (e.g. determination of new standards that can be achieved with new technology)	[7, 14, 16, 17, 36]	X	
Disruptions	Disruptions in the supply chain (e.g. the COVID-19 crisis) push SMEs to adopt technologies and change their BM to cope with them	[12–14, 28, 36–45]	X	X
Technology turbulence	Rate of technological advancement within an industry influence the decision to change the BM [46]	[46–48]		X

Organizational drivers	Description	References	Classification	
			TA	BMI
Top management support	Support of owner/manager in introducing new technology and/or changing the business	[1, 4, 10, 12, 49–51]	X	X
Perception of market opportunities or changes	SMEs proactively conduct technological transformation to attend to perceived market opportunities or changes	[14, 35, 49, 52]	X	X
Strategic orientation	Orientation towards strategic decisions enables the implementation of technology into BMs	[5, 47, 48, 53]	X	X
Innovation activities	A company undertakes activities to add value to its products and services	[32, 47, 48]		X

References: [1] Hussein et al. (2019); [2] Pavic et al. (2007); [3] Gatautis and Vitkauskaite (2009); [4] O'Reilly and Ugray (2010); [5] Gayen et al. (2016); [6] Moeuf et al. (2018); [7] Groot et al. (2019); [8] Mohapatra and (2019); [9] Apostolov and Coco (2021); [10] Mujahed et al. (2022); [11] Kraft et al. (2021); [12] Hussain et al. (2021); [13] Akpan et al. (2022); [14] Ludin et al. (2022); [15] Saáry et al. (2022); [16] Bracci et al. (2022); [17] Cinjarevic et al. (2021); [18] Lim and Baharudin (2013); [19] Nichifor et al. (2022); [20] Chen et al. (2018); [21] Cranmer et al. (2021); [22] Ahmad et al. (2018); [23] Chonsawat and Sopadang (2020); [24] Yang et al. (2019); [25] Norris (2020); [26] Kim et al. (2008); [27] Brown and Lockett (2004); [28] Gregurec et al. (2021); [29] Jutla et al. (1999); [30] Chan and Chung (2002); [31] Zhang et al. (2015); [32] Pucihar et al. (2019); [33] Yang et al. (2022); [34] O’Toole (2003); [35] Müller et al. (2018); [36] Subriadi and Wardhani (2022); [37] Priyono et al. (2020; [38] Akpan et al. (2021); [39] Paoloni et al. (2022); [40] Giotopoulos et al. (2022); [41] Ragazou et al. (2022); [42] Lu et al. (2022); [43] Khurana et al. (2022); [44] Zamani et al. (2022); [45] Musa et al. (2022); [46] Molina-Castillo et al. (2022); [47] Gatautis et al. (2019); [48] Bouwman et al. (2018); [49] Alexander et al. (2003); [50] Garzella et al. (2020); [51] Müller et al. (2021); [52] Loon and Chik (2019); [53] Yousaf et al. (2021)

Regarding technological drivers, the relative advantage (also referred to as potential/perceived benefits) is one driver often cited in the literature. Some relative advantages expected by SMEs are cost reduction, operational efficiency, and increased sales. Moreover, recent studies claim that technologies may positively impact sustainability; for instance, digital technologies may enable improvements in carbon emission, material consumption, and waste reduction (Saáry et al. 2022). From the papers analyzed, most of the benefits mentioned are potential (i.e. not empirically confirmed by the studies).

As researchers define relative advantage differently, relative advantage and perceived usefulness are often used interchangeably in literature (Hussain et al. 2021). Perceived usefulness is also related to the benefits expected but focused on enhancing job performance (Hussein et al. 2019), while relative advantage is a broader concept. Ease of use is also a technological driver, which is expected since SMEs generally do not adopt complex systems (Brown and Lockett 2004).

SMEs' technological structure and previous experience also impact the decision to adopt technology: the greater the compatibility between enterprise policy and technology, the greater the likelihood of adopting the new technology (Cinjarevic et al. 2021). Moreover, as SMEs lack financial and technological resources, technology readiness is also a predictor of technology adoption (Gayen et al. 2016) since adopting certain technologies requires infrastructure and skills (Hussain et al. 2021).

Regarding environmental drivers, the support of a trusted party is often mentioned as a driver for SMEs to engage in technology assimilation and/or BMI since cooperation with entities is seen as a way to mitigate the risks related to innovation (Yang et al. 2019; Brown and Lockett 2004). Trusted parties can be technology providers, government, cooperatives, industry associations, or private or public institutions.

Studies emphasize the role of technology providers as a relevant trusted parties supporting technological transformation in SMEs. Several papers consider the provider perspective or provider support aspects central in their studies (e.g. Groot et al. 2019; Kim et al. 2008; Mohapatra and Thakurta 2019). According to Kim et al. (2008), some of the reasons for using a technology provider are: (i) predictable costs; (ii) guaranteed performance levels; (iii) Free up staff to focus on internal issues; (iv) providers have expertise; (v) quicker implementation; (vi) Automatic upgrades; (vii) Guaranteed uptime; (viii) Lack of internal resources; (ix) Security concerns; (x) Try out the technology before buying.

The existence of feasible BM for technologies is discussed in the literature based on the user or provider perspectives. For users, feasible BMs support the decision to adopt technology in SMEs (Jutla et al. 1999). For providers, the perception of new opportunities supports in creation of new value propositions that meet SMEs' needs. For example, Groot et al. (2019) state that the limited time window of Climate Smart Agricultural (CSA) technologies constrains SMEs' technology adoption. In this regard, the authors suggest that a concrete action to mitigate this constraint would be leasing machinery in other districts. Moeuf et al. (2018) provide another example of a barrier related to technological characteristics: some industry 4.0 technologies are expensive with a long return on investment, and new BMs for SMEs are required.

Competitive pressure is related to the industry sectors in that SMEs are operating (Gatautis and Vitkauskaite 2009), and those SMEs that operate in a competitive environment are more likely to adopt new technologies (Hussain et al. 2021) and change their BM (Pucihar et al. 2019). SMEs' customers and/or suppliers can exert external pressure to adopt a particular technology, given contracts with specific customers (Apostolov and Coco 2021) and pressure from larger partners (O’Toole 2003).

Government regulations can also drive technology adoption (directly or indirectly). For example, Groot et al. (2019) state that the Punjab government began enforcing the ban on burning rice and wheat stubble in farmlands, which can drive the adoption of CSA technologies. Another environmental factor that drove technological transformation in SMEs is disruptions, such as the COVID-19 crisis, which have received increasing academic attention within the last 2 years (e.g. Song et al. 2022; Musa et al. 2022; Akpan et al. 2022). In this context, technology was a survival source during the pandemic, as companies adopted digital technologies to maintain their operations (Akpan et al. 2021; Priyono et al. 2020). Technology turbulence is an environmental driver related to rapid technological development within an industry that motivates to engage in BMI (Bouwman et al. 2018).

Organizational drivers are mainly related to top management actions and vision. Firstly, top management can provide support and pursue strategic orientation toward technological transformation. SMEs can also be internally motivated to adopt technologies when the owner/manager perceives market opportunities or changes in the market (e.g. changes in consumer behavior) that new technology can address (Alexander 2003; Ludin et al. 2022). According to Müller et al. (2018), whether the company is internally motivated and/or externally pressured towards adopting new technology impacts which BM elements are innovated. Finally, the conduction of innovation activities can also play a role in decisions related to BMI, such as technology adoption.

#### Barriers to technological transformation

The barriers to technological transformation can be related to its decision (i.e. the decision to adopt a technology or change the BM by using a technology) or its process (i.e. transforming the business to exploit new technology). Indeed, the barriers vary according to the stage of technology assimilation (Gatautis and Vitkauskaite 2009) and BMI (Santa-Maria et al. 2022), consequently varying according to the stage of technological transformation. The barriers identified are shown in Table 6.

**Table 6:** Barriers to technological transformation

Technological barriers	Description	References	Classification	
			TA	BMI
Security concerns	Technology needs to accomplish security aspects, such as confidentiality and data privacy [1]	[1–19]	X	
High cost	Technology costs can include installation, training, upgrading, and hiring people	[1–6, 8, 11, 17, 20–30]	X	
Complexity	SMEs have difficulties in mastering the complexities of some technologies [15]	[2, 11, 15, 18, 20, 31]	X	
Interoperability/ Integration	Ensuring technology interoperability/integration with existing systems, standards, and interfaces within and across companies [6]	[3–7, 11, 15, 16, 32]	X	

Environmental barriers	Description	References	Classification	
			TA	BMI
Poor socio-economic situation	Context characterized by poor education, infrastructure, and lack of government support [35]	[25, 29, 33–36]	X	X
Unfavorable regulatory landscape	Policies that do not attach importance, urgency, or present bureaucracy to innovations	[2, 6, 12, 13, 16, 17, 28, 29, 36, 37]	X	X
Lack of access to finance	Difficulties in accessing finance or financial support do not match SMEs' needs	[12, 17, 22, 28, 29, 36, 37]	X	X
Problems in partnerships	Lack of business partners or partners' problematic behaviors. Complexities in managing partnerships	[6, 18, 28, 29, 37–41]	X	X
Lack of customer acceptance/demand	Lack of customer acceptance/demand regarding technology use or its related products/services	[3, 15, 28, 41–45]	X	X

Organizational drivers	Description	References	Classification	
			TA	BMI
Lack of awareness concerning technology	SMEs can be unaware of new technologies	[1, 3, 6, 11, 17, 46, 47]	X	
Lack of awareness concerning BMI	Unawareness about BMI, how to do it, and BM related to specific technologies [48]	[5, 6, 15, 19, 30, 46, 48, 49]		X
Lack of resources	Lack of resources in terms of people, time, and finances	[5, 6, 11, 17–19, 24, 26, 37, 42, 50–52]	X	X
Lack of skilled workers Difficulty in hiring skilled workers	SMEs usually do not have skilled workers and face difficulties in hiring or finding skilled workers	[2, 8, 15, 17, 18, 22, 28, 29, 36, 42, 48, 53, 54]	X	
Mistrust	Mistrust that a particular technology will provide benefits	[6, 11]	X	
Resistance to change	Employees resist using technology and changing their ways of working	[3, 15, 17–19, 29, 32, 42]	X	X
SMEs' conservative profile	SMEs are more reactive than proactive in innovating	[5, 6, 8, 29]	X	X
Lack of strategy	Lack of strategy and planning leads to difficulties in achieving the potential benefits of technology	[5, 39, 50, 54, 55]	X	X
Lack of skills	Lack of skills to manage the technology assimilation and/or business transformation	[3, 6, 11, 28–30, 32, 39, 41, 42, 48, 55, 56]	X	X
Difficulty in implementing changes in the current BM	Difficulties in balancing the current BM with new BM practices when engaging in business transformation	[2, 6, 11, 17, 55, 57, 58]		X
Challenges for creating, delivering, and capturing value	Difficulties in deploying innovative products or services related to a technology effectively. The difficulties can also be in terms of delivery and capture of the new value proposition	[39, 41, 54, 59, 60]		X
Previous bad experiences with technology	Previous bad experiences with technology inhibit the intention to adopt and use new technologies	[2, 11]	X	
Dependency on external expertise	SMEs depend on external parties (e.g. technology providers) to acquire knowledge and skills	[42, 61]	X	
Difficulty in entering the market	Big players dominate markets	[25, 39, 60]		X
Leadership issues	The managers are unwilling to be responsible for technology assimilation or BMI or lack understanding of the required actions	[11, 51, 60]	X	X
Short term vision	SMEs generally have a short-term orientation, and the benefits of technologies may take time	[5, 62]		X

References: [1] Mohapatra and Thakurta (2019); [2] Rozmi et al. (2020); [3] Westerlund (2020); [4] Venkatachalam et al. (2014); [5] O’Toole (2003); [6] Gatautis and Vitkauskaite (2009); [7] Jhang-and Chang (2017); [8] Lim and Baharudin (2013); [9] Hussein et al. (2019); [10] Karunagaran et al. (2019); [11] Pavic et al. (2007); [12] Akpan et al. (2021); [13] Bracci et al. (2022); [14] Gavrila and Ancillo (2021); [15] Birkel et al. (2019); [16] Juszczyk and Shahzad (2022); [17] Kumar et al (2022); [18] Lu et al. (2022); [19] Pfister and Lehmann (2022); [20] Moeuf et al. (2018); [21] Cranmer et al. (2021); [22] Ingaldi and Ulewicz (2020); [23] Hao et al. (2018); [24] Kim et al. (2008); [25] Mkansi et al. (2019); [26] Chan and Chung (2002); [27] Pomffyová et al. (2017); [28] Fanelli (2021); [29] Indrawati et al. (2020); [30] Musa et al. (2022); [31] Brown and Lockett (2004); [32] Teoh et al. (2022); [33] Hussain et al. (2021); [34] Mujahed et al. (2022); [35] Akpan et al. (2022); [36] Groot et al. (2019); [37] Alshareef and Tunio (2022); [38] Kollmann and Häsel (2008); [39] Apostolov and Coco (2021); [40] Bootz et al. (2022); [41] Reim et al. (2022); [42] Bourdon and Jaouen (2016); [43] Coreynen et al. (2017); [44] Huynh (2022); [45] Jutla et al. (1999) [46] Azevedo and Almeida (2021); [47] Nichifor et al. (2022); [48] Ibarra et al. (2020); [49] Chiarvesio et al. (2022); [50] Sell et al. (2019); [51] Brink (2017); [52] Matenga and Mpofu (2022); [53] Norris (2020); [54] Chen and Zhang (2015); [55] Trstenjak et al. (2022); [56] Balocco et al. (2012); [57] Alexander et al. (2003); [58] Loon and Chik (2019); [59] Shin (2017); [60] Yang et al. (2019); [61] Priyono et al. (2020); [62] Isensee et al. (2020)

Concerning technological barriers, security concerns relate to data-driven technologies (e.g. ICT) and are considered a barrier to adoption (Mohapatra and Thakurta 2019) and to the intention to continue using the technology (Hussein et al. 2019). SMEs are concerned about risks associated with new technologies, such as keeping privacy and confidential corporate data (Mohapatra and Thakurta 2019), transaction security (Gatautis and Vitkauskaite 2009), and losing data. Indeed, data and information are some of the most valuable assets for companies (Lim and Baharudin 2013).

The high cost is also a barrier to adopting and evolving the technology assimilation process. SMEs have limited possibility to invest in technologies; thus, their decision is significantly influenced by cost. Two studies have shown the preference of SMEs to adopting cheaper technologies: (i) Moeuf et al. (2018) concluded in their literature review that SMEs exploit low-cost technologies of industry 4.0; (ii) Mohapatra and Thakurta (2019) concluded through a survey with Indian SMEs that the pricing tariff is the most important factor in choosing a cloud computing provider.

SMEs generally do not adopt complex systems (Brown and Lockett 2004; Rozmi et al. 2020). For instance, Brown and Lockett (2004) state that SMEs appear comfortable with low-complexity applications (e-mail) but present limited or no engagement with high-complexity applications (e-marketplaces). According to the authors, SMEs would not adopt complex applications without substantial support. Another technological barrier is the difficulty of ensuring the technology interoperability with existing systems (Gatautis and Vitkauskaite 2009) within and between companies.

Three environmental barriers are related to institutional context: poor socio-economic situation, unfavorable regulatory landscape, and lack of access to finance. Poor-socio economic situation is an unfavorable local context primarily related to developing countries, which are not on par with developed countries. Developing countries face additional challenges regarding technological transformation, which may be linked to the low level of internet penetration, dysfunctional educational systems, poor infrastructure and malfunctioning political and economic systems (Akpan et al. 2022).

The unfavorable regulatory landscape restricts technological transformation due to: policies that do not attach importance or urgency to technologies (Rozmi et al. 2020), lack of government support (Indrawati et al. 2020), differences in legal and regulatory environments in cross-border transactions (Gatautis and Vitkauskaite 2009), and lack of regulation for new technology (Bracci et al. 2022). For example, recent studies show how the lack of regulation hampers the use of blockchain technologies (Alshareef and Tunio 2022; Juszczyk and Shahzad 2022). Moreover, SMEs can be challenged by the lack of access to finance, especially for new organizations (Alshareef and Tunio 2022). Access to finance can be challenging due to long and arduous application processes, existing subsidies that do not match SMEs' reality (Groot et al. 2019), or predatory lending (Akpan et al. 2021).

Problems in partnerships can be in terms of (i) lack of partners, (ii) behavior of partners, and (iii) relationship management. According to Kollmann and Häsel (2008), alliances, in general, can present severe conflict and opportunism during the entire collaboration time because integrating different BMs is inherently risky. Still, according to the authors, the risks are related to partner identification and subsequent project execution. Companies need to understand (i) if the collaboration is strategic and (ii) if the organizational structure and company culture of partners fit (Kollmann and Häsel 2008).

Certain technologies and BMI require collaboration to leverage benefits and fulfil functions; thus, partnership problems may be even more problematic for those cases. For example, Reim et al. (2022) identified three BMI challenges (in the case of internationalization) related to partnerships: (i) difficulties in identifying, attracting, and engaging with international partners, (ii) lack of suitable international partners, and (iii) difficulties in building trust with partners. Another example is blockchain technology which requires a solid and extensive network to enable SMEs to fully capitalizing on its advantages (Alshareef and Tunio 2022).

The lack of customer acceptance/demand is an external resistance to the technology use or related products and/or services. For example, Coreynen et al. (2017) describe the product-service systems offerings, which present a cultural shift for customers (e.g. not owning products), which can lead to a non-acceptance. Reim et al. (2022) identify different challenges related to customer acceptance in the case of internationalization, such as lack of suitable customers, limited market information, challenging market and visibility with foreign customers, new customer acquisition, and fluctuation in demand. Moreover, SMEs present difficulties in producing new offerings (Coreynen et al. 2017) and commercializing technology products (Shin 2017). In fact, SMEs may face difficulties in creating new value propositions and also in delivering and capturing value from technological transformation (Reim et al. 2022).

Regarding organizational barriers, the knowledge and perception of the owner-manager play a central role in decisions of technological transformation. The owner-manager can be unaware of new technologies available (Azevedo and Almeida 2021), leading to non-adoption. The owner-manager perception' affects their intention to adopt technology, and previous bad experiences with technology or mistrust (especially for new technologies that do not present clear evidence of their benefits, with uncertain profitability) also represent an adoption barrier.

If not from a technology background, the owner-manager may be unaware of how to exploit a technology (O’Toole 2003) or how to do BMI (e.g. process and techniques). Another difficulty managers face is taking responsibility for the changes (Yang et al. 2019) or understanding the changes required by new technology. SMEs generally do not possess highly skilled workers, and depending on the level of expertise required, it can be challenging to hire qualified workers due to the scarcity of these professionals and the limited career prospects offered by SMEs (Birkel et al. 2019). Therefore, finding and retaining those professionals can be challenging for SMEs (Lu et al. 2022). For example, Birkel et al. (2019) state that industry 4.0-related technologies will need hiring specialists with unique skills (e.g. computer science, programming, data security), which is challenging for SMEs because these specialists are scant, and SMEs cannot afford them in many cases.

SMEs' employees may also resist changes imposed by new technology due to a lack of specific technical knowledge, difficulty adapting, fear of job loss, or loss of recognition/responsibilities (Birkel et al. 2019). According to Bourdon and Jaouen (2016), changing employees’ resistance can be easily managed in large companies, but in SMEs, there is a magnification effect; the relative impact of an individual is multiplied (one employee out of ten means that 10% of workforce resists change). The employees may also not be committed to change due to additional workloads required, as reported by one of the case studies of Teoh et al. (2022) of an SME that adopted digital BMI.

SMEs are characterized by a lack of resources such as financial, time, and competence (Sell et al. 2019). Given this context, SMEs are more conservative and aversive to risks and can also have a short-term vision. When short-term benefits do not appear, a “wait-and-see” attitude appears to prevail, which may be attributed to an absence of strategic planning (O’Toole 2003). The lack of resources restricts BMI (Reim et al. 2022) and technology assimilation (Mohapatra and Thakurta 2019), restricting the pace of technological transformation in SMEs. The lack of resources also implies difficulties in entering new markets (Yang et al. 2019).

The difficulty in implementing changes in the current BM is also an organizational barrier. For example, Alexander (2003) states that larger firms would expect to have an easier time moving to e-commerce since they have a more departmentalized infrastructure, and management functions would not include dealing with day-to-day operations, marketing, supplier relationships, and contract negotiations. Moreover, SMEs may also be stuck in the “old way of doing things” (Pavic et al. 2007) or that existing BM is incompatible with the new structures (Kumar et al. 2022).

Finally, SMEs generally depend on external expertise for most of the technical expertise (O’Toole 2003). Alexander (2003) describes the case of a small company transitioning to e-commerce that was first dependent on the technology providers and after the owner-manager developed expertise in-house. Priyono et al. (2020) also mentioned a case in which the company relied on partners for its digital functions. The authors state that this is a temporary solution, and for sustainable competitive advantage, they need to develop digital capability in-house.

#### Enablers to technological transformation

The summary of enablers identified in the literature is presented in Table 7. Interestingly, all the enablers identified can be applied to BMI and technology assimilation.

**Table 7:** Enablers for technological transformation

Organizational enablers	Description	References	Classification	
			TA	BMI
Top management support	Top management creates a supportive innovation environment and provides the required resources for TA and BMI	[1–7]	X	X
Commitment of resources/investments	Commitment of resources entailing budgets, human capabilities, and time for innovation	[1, 4, 7–13]	X	X
Organizational culture favorable to technological transformation	Shared values, ideologies, philosophies, assumptions, beliefs, expectations, attitudes, and norms determine people's behavior [15]	[3, 8, 14–16]	X	X
Companies’ capabilities	Different capabilities are required for technological transformation, such as dynamic capabilities, networking, managerial, and technological capabilities	[3, 6, 10, 12–31]	X	X
Strategic alignment	Strategic alignment of business strategy and technology strategy	[3, 4, 7, 14, 24, 32–34]	X	X

Environmental enablers	Description	References	Classification	
			TA	BMI
Partnerships	SMEs need to collaborate with other organizations for different purposes, such as accomplishing activities in the supply chain, acquiring technologies, equipment or skills, funding, and collaborating in product development and innovation	[4, 10, 12, 14, 20, 27, 33, 35–37]	X	X
Support of a trusted party	External support of trusted parties for conducting innovation activities	[7, 38–44]	X	X

References: [1] Alexander et al. (2003); [2] Hussein et al. (2019); [3] Ibarra et al. (2020); [4] O’Toole (2003); [5] Müller et al. (2021); [6] Garzella et al. (2020); [7] Teoh et al. (2022); [8] Bouwman et al. (2019); [9] Moeuf et al. (2018); [10] Apostolov and Coco (2021); [11] Chen and Zhang (2015); [12] Westerlund (2020); [13] Paiola et al. (2022); [14] Priyono et al. (2020); [15] Isensee et al. (2020); [16] Khattak (2022); [17] Xie et al. (2022); [18] Song et al. (2022); [19] Kim et al. (2018); [20] Kollmann and Häsel (2008); [21] Liu et al. (2021); [22] Loon and Chik (2019); [23] Rozmi et al. (2020); [24] Soluk and Kammerlander (2021); [25] Tali et al. (2021); [26] Vrontis et al. (2021); [27] Kassim et al (2016); [28] Fanelli (2021); [29] Gerlitz (2016); [30] Herve et al. (2020); [31] Coreynen et al. (2017); [32] Chen et al. (2018); [33] Chan and Chung (2002); [34] Pfister and Lehmann (2022); [35] Wu et al. (2022); [36] Gutierrez-Leefmans and Holland (2019); [37] Mkansi et al. (2019); [38] Venkatachalam et al. (2014); [39] Groot et al. (2019); [40] Brown and Lockett (2004); [41] Balocco et al. (2012); [42] Gatautis and Vitkauskaite (2009); [43] Yang et al (2019); [44] Huynh (2022)

Top management plays an essential role in creating the conditions for technological transformation since they act on (i) the definition of innovation strategy; (ii) the commitment of resources for technological transformation; (iii) the culture towards technological transformation; and (iv) alignment of operational and strategic actions. The innovation strategy needs to encompass the conditions for using the technology and change the business (activities, value proposition, partnerships, the balance of revenue and cost) according to marketing opportunities. Top management should provide guidance, supervision, and training, especially in the first moment of technology introduction, to ensure that the employees can operate with new technology and do not commit repetitive mistakes (Teoh et al. 2022).

SMEs need to consider strategies for: acquiring knowledge and resources regarding the new technology (Groot et al. 2019; Apostolov and Coco 2021); value creation according to the technology (Chan and Chung 2002; Coreynen et al. 2017); technology alignment, i.e. vision, objective, structures (Venkatachalam et al. 2014); enable functions to deliver value, e.g. co-design with customers, integrating distribution channels, outsource weaker functions (Chan and Chung 2002); and enable value proposition and revenue model (Gutierrez-Leefmans and Holland 2019).

In general, partnerships are often part of SMEs’ strategy for technological transformation, given their lack of resources. These partnerships can be for acquiring new technology and knowledge; for example, cooperatives offer relatively cheap loans for renting and/or purchasing technology (Groot et al. 2019). Partnerships can be aimed at satisfying niche markets together; for instance, a company’s R&D can collaborate with a partner’s manufacturing department to develop a new product and manufacture it cheaply (Chan and Chung 2002). Other examples are companies with no global entry experience that partner with experienced global market players (Shin 2017).

External collaboration can include (i) partnerships among companies to accomplish business functions within the value chain or (ii) external support. In the first case, connecting ecosystem partners entails risks such as interdependence and coordination challenges, necessitating BMs' alignment with the value network and strategic risk management (Birkel et al. 2019). External support involves parties such as the government, technology providers, and universities supporting innovation activities. According to Venkatachalam et al. (2014), the scope of provider support can go beyond adoption and implementation challenges, including technology orientation, operation alignment, and strategic alignment. The SMEs can also collaborate with customers to better address their needs (Westerlund 2020; Coreynen et al. 2017).

Resource allocation is critical for technological transformation (Chen and Zhang 2015; Westerlund 2020). In this regard, although studies state that investments in technology assimilation and BMI have a positive impact on technological transformation success (Alexander 2003; Bouwman et al. 2019; Chen and Zhang 2015), few practical orientations are given on how to decide on those investments given SMEs’ lack of resources.

The owner/manager's actions influence the culture towards technological transformation in SMEs. In this regard, their actions need to create a positive environment for technological transformation, which include training in the new way of doing things and accepting the new technology. According to Chen et al. (2018), technology, people, and organization must be appropriately aligned to benefit from technology.

Finally, the success of the technological transformation is also shaped by the companies' capabilities (e.g. technological, managerial, network, and dynamic capabilities), which depend on their context. In this regard, SMEs can achieve outcomes by combining different capabilities (Priyono et al. 2020; Ibarra et al. 2020).

#### Control variables—Size, age, industry, and technology type

Papers consider industry sector (Del Giudice et al. 2021; Qu et al. 2021), age (Wang et al. 2020; Denicolai et al. 2021), and size (Wang et al. 2020; Denicolai et al. 2021) as control variables. The industry sector influences the innovation and competitive conditions in the environment (Del Giudice et al. 2021), influencing the technologies adopted and BMI practices. Moreover, the age of a company influences acquired tangible and intangible resources (Del Giudice et al. 2021), influencing technological transformation practices. For example, the BMI phenomena in established firms differ substantially from newly created companies (Ibarra et al. 2020). Authors also claim that the older the SME, the less likely it will adopt certain technologies (Pavic et al. 2007; Rozmi et al. 2020).

Company size influences the company's resources, determining the possibilities for SMEs to adopt technologies and the paths for transformation. In this regard, technologies recognized as “new” for SMEs can be standard for larger companies, and technologies that are feasible for medium companies may not be feasible for small ones (Olson 2018). Finally, technology type also influences technological transformation since technological characteristics play a role in the level of BMI changes (incremental or radical) (Bouwman et al. 2018; Leelasantitham 2020; Lokshina et al. 2018). For example, Bouwman et al. (2018) concluded that social media and big data affect BMI differently. According to the authors, big data has a broader impact than social media because social media usage is more focused on channels, whereas big data can affect companies in all of their core activities as well as the activities of their key partners.

#### Process of technological transformation

Few papers focus on the process of technological transformation by describing SMEs that successfully transformed or intend to transform (e.g. Alexander 2003; Liao 2004), and the studies fail to provide details about SMEs that have discontinued technology use or failed in technological transformation. The case studies focus on the actions and changes made during the transformation process but generally do not describe the phases or tools for technology assimilation, BMI, or technological transformation. Exception for the longitudinal case study conducted by Paiola et al. (2022) regarding the process of IoT-driven BMI. The authors state that the process is incremental and delimitate the phases of BMI: inception, experimentation, and replication. However, a model combining technology assimilation and BMI is still lacking.

Few studies present more insights into the transformation process by presenting paths (e.g. Coreynen et al. 2017; Shin 2017; Priyono et al. 2020; Teoh et al. 2022). These studies analyze the current companies' resources and capabilities, the barriers to technological transformation, and the strategies to overcome these barriers. For example, Priyono (2020) present different case companies which responded differently to digitalization: SMEs with a high level of digital maturity accelerated the transition toward digitalized firms; SMEs with low digital maturity that only digitize their sales functions; and SMEs with low digital literacy that outsource their digital functions to partners. Recent studies propose the evaluation of the current situation of SMEs before transformation by assessing the readiness factor (Trstenjak et al. 2022) and the maturity level (Petzolt et al. 2022). These studies support strategic planning for technological transformation, enabling a better understanding of the transition.

Studies suggest that SMEs without external support and previous knowledge regarding technologies experience a gradual transformation process, such as a step-by-step (Shin 2017; Mohapatra and Thakurta 2019; Müller et al. 2018), adopting technologies in one function of the company (Priyono et al. 2020), or outsourcing (Chan and Chung 2002). In this sense, outsourcing requires managing the relationship between the firms and partners (Priyono et al. 2020) while acquiring and integrating knowledge will be shaped by the firms' managerial capacities (Apostolov and Coco 2021).

Studies suggest the importance of providers in the technological transformation process (e.g. Brown and Lockett 2004; Groot et al. 2019; Alexander et al. 2003), especially when the technology is complex or the company does not possess the required technical capabilities, with providers also supporting in BMI process (e.g. Venkatachalam et al. 2014; Brown and Lockett 2004). In this regard, the analysis of the papers suggests an overlap of BMI and technology assimilation, but the papers do not present details on this overlapping or on how to integrate these processes.

#### Interrelatedness between technology assimilation and BMI

Smajlović et al. (2019) indicate two relationships between technology assimilation and BMI (i) technology assimilation that drives BMI, and (ii) BMI mediates the relationships between technology assimilation and the company’s performance, which were also identified in the papers analysed. Firstly, technology assimilation can drive BMI, given its requirements/dynamics (papers on the foci 1, 2, 3, and 4 – Table 4). For example, Priyono (2020) describes SMEs that digitalized the sales function and needed redesigning distribution and sales channels. Alexander (2003) describes a small company that adopted e-commerce and needed to change key activities and resources (e.g. developing IT function, web developer), and customer relationships (before in person and afterwards online). Pucihar et al. (2019) state that new information technologies enable the design of innovative and new BMs, which often disrupt existing industries and markets.

Secondly, papers in focus 6 relate to the second relationship (BMI mediates the relationships between technology assimilation and the company’s performance). In this relationship, BMI determines the nature of complementarity between BMs and paths for monetization. This complementarity includes value mechanisms such as value offer (the value embedded in the product/service offered to the customer), value creation (identifying customers and how they are engaged) and value capture (how value is delivered and monetized) (Müller et al. 2018). In sum, BMI connects technology and value by producing products or services that customers will pay for, and selecting the right technology and customer segments is part of BMI's strategy.

Finally, we identified a third relationship BMI as a precursor of technology assimilation (papers in focus 5), which is not commonly addressed in the literature. This relationship is focused on enabling SMEs to acquire/use technologies that were not previously feasible for these companies. Although this relationship is not often discussed in the literature, it is a topic that makes sense in the SMEs' context, given their lack of resources.

#### Outcomes of technological transformation

The outcomes of technological transformation can be discussed in terms of (i) the level of technology assimilation, (ii) the level of BMI, and (iii) performance outcomes. The level of technology assimilation can be evaluated in terms of the stage of technology assimilation within a company. For example, Fichman and Kemerer (1997) employ a six-stage to classify the level of technology assimilation: (i) awareness, (ii) interest, (iii) evaluation/trial, (iv) commitment, (v) limited deployment, and (vi) general deployment. In the case of digital technologies, authors classify the level of technology assimilation in terms of the extent to which technology adoption has spread in three areas: organization and management, marketing and sales, and production (Moeuf et al. 2018; Sabatini et al. 2022).

The level of BMI refers to the extent to which BM elements have been changed (Sabatini et al. 2022), ranging from a few changes to an entirely new BM. Authors propose different ways to classify the level of BMI based on criteria such as novelty and scope (Foss and Saebi 2017) or the number of BM elements impacted – also referred as complexity of BMI (Sabatini et al. 2022).

The level of technology assimilation and BMI are connected, and the studies discuss the level of technology assimilation in terms of the extent to which technologies affect SMEs' BMs (Sabatini et al. 2022). For instance, Westerlund (2020) claims that internationally-oriented online SMEs differ from domestically-oriented ones in terms of, among other things, a higher degree of use of information systems. In general, the studies assert that SMEs achieved a low level of technology assimilation and BMI (e.g. Moeuf et al. 2018; Müller et al. 2021; Bracci et al. 2022; Reuschke and Mason 2022; Akpan et al. 2022).

This low transformation level may occur for more recent technologies that SMEs have just started to acquire/use, and these technologies take time to be assimilated by SMEs (Moeuf et al. 2018; Müller et al. 2021). The low level of technological transformation outcomes may also be related to the concentration of papers on the factors, especially barriers (which inhibit SMEs from achieving higher levels of technology assimilation and BMI), with some studies dedicated to those barriers (e.g. Jhang and Chang 2017; Birkel et al. 2019; Fanelli 2021; Indrawati et al. 2020; Kumar 2022). There is a concentration of papers in the first phases of technology assimilation, such as adoption (e.g. Bracci et al. 2022; Mujahed 2022; Kumar 2022). The low level of adoption or assimilation leads to a low level of BMI. Finally, a few papers assess the performance outcomes expected from the technological transformation in SMEs. Next section presents a discussion of the findings.

## Discussion

Based on our findings, we suggest some possible explanations for the fragmentation of the literature on technological transformation in SMEs. Firstly, this field is young, as shown in descriptive analysis (most studies were published between 2017 and 2022). Secondly, the context-dependence of technological transformation (in terms of technology, industry, age, and firm size) hampers its general conceptualization, with a concentration of papers in some technological contexts. Thirdly, the research streams are also fragmented individually as technology assimilation and BMI can be discussed in terms of process or outcome, resulting in a complex combination of the fields due to the high level of abstraction.

To improve the understanding of technological transformation in SMEs, we conduct an initial merge of technology assimilation and BMI fields in the context of introducing a technology by (i) classifying the papers' foci; (ii) identifying, unifying, and positioning the factors; (iii) identifying what is stated about the process, control variables and outcomes; and (iv) providing a conceptual framework.

We identified six main papers' foci, the most prominent being factors. The concentration of papers in analyzing factors was expected since existing literature reviews on technology assimilation and BMI in SMEs also focus on factors (Ahmad and Siraj 2018; Hidayat et al. 2020). Moreover, as SMEs lag behind larger companies in adopting technologies, it is not surprising that papers focus on drivers to engage in technological transformation and the barriers that inhibit SMEs in this process. Moreover, from the classification of the factors, we demonstrate that although there are specific factors for technology assimilation and BMI, there are many common factors (especially enablers). The overlapping factors signal the holistic nature of technological transformation (e.g. partnership problems represent a barrier to technology assimilation and BMI).

The analysis of the factors depicts two crucial agents for technological transformation: the owner/manager and technology providers. Owner/managers are central to decision-making and creating conditions for technological transformation, which depends on their background and experience. Technology providers can support creating the conditions for using new technologies and also support SMEs in the strategic alignment of their BMs. The fact that providers need to change their BMs (not only concentrating on selling and installing technology) to meet SMEs' needs is in line with the integrative technological transformation perspective on introducing technology.

Regarding the process of technological transformation, the analysis highlights the need for external support, especially in cases where technology is complex or new and the SME does not possess specific capabilities (e.g. technical, financial, marketing). Moreover, there is a lack of conceptualization of the technological transformation process that may be linked to its context dependence. This lack of conceptualization challenges SMEs since technological transformation combines two already challenging processes with interrelationships.

The interrelationships between BMI and technology assimilation are not generally addressed in the literature, although the overlapping of the processes is suggested. From the analysis of the papers, we identified three relationships: (i) technology assimilation that drives BMI, (ii) BMI that mediates the relationships between technology assimilation and a company’s performance, and (iii) BMI as a precursor of technology assimilation. The last relationship is not usually addressed in the literature but emerged from the analysis since technology acquisition can be problematic for SMEs.

**Fig. 2:**
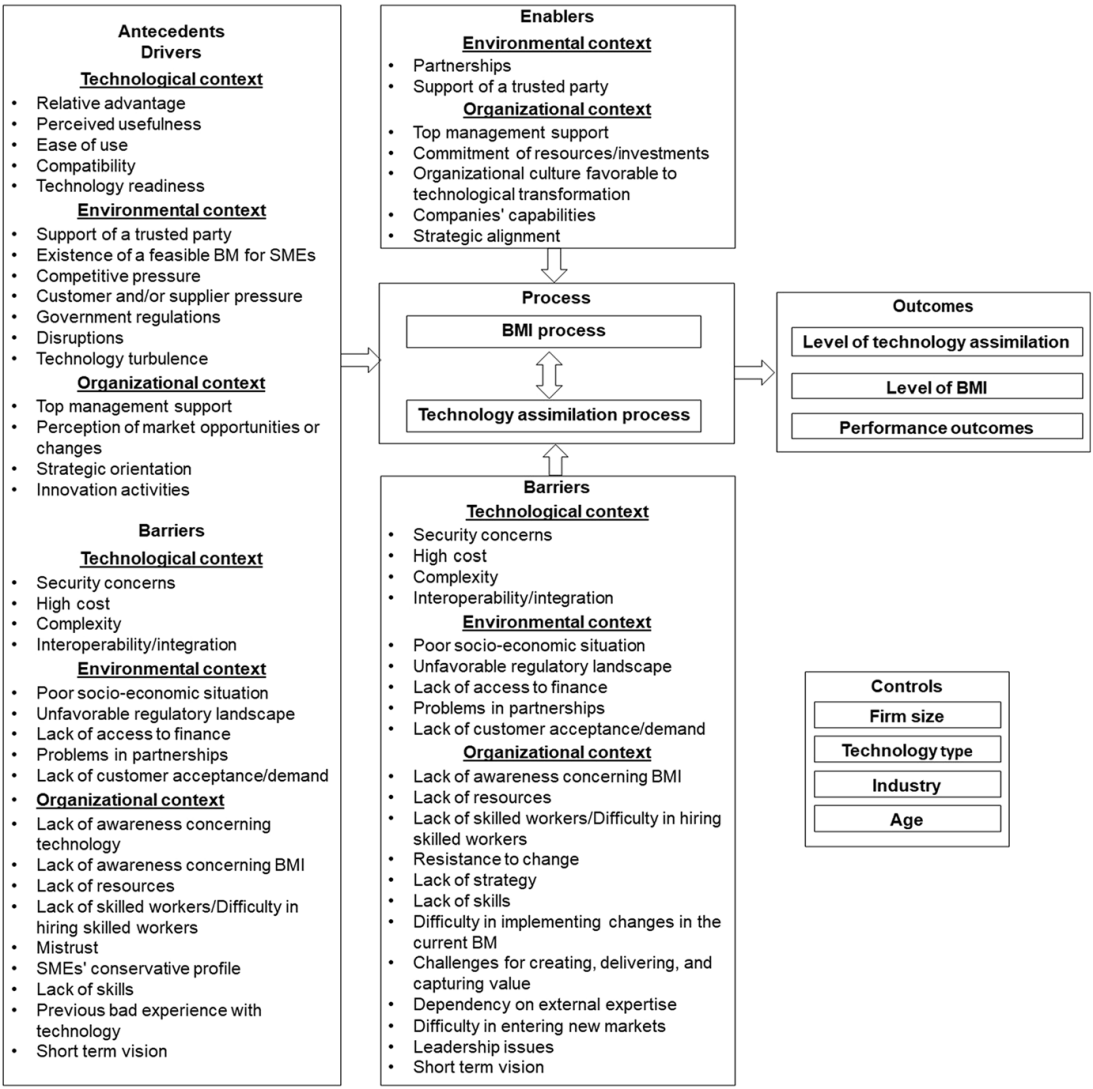
Technological transformation framework

The studies generally show a low level of transformation in terms of technology assimilation and/or BMI, and few studies evaluate the performance outcomes of technological transformation. This fact may be linked to the recentness of the papers and to SMEs' characteristics that lag in the technology adoption or experiment with a gradual transformation process compared to larger firms, which may result in the lack of studies on performance outcomes or the low level of outcomes reported. Finally, based on the content analysis, we derive the technological transformation framework (Fig. 2).

To build this framework, we considered the overview of technological transformation: from its initiation to its outcomes. The drivers identified (presented in detail in Sect. 3.2.2) are positioned at the beginning of the process, as they are the triggers for introducing new technology and/or changing the business with the new technology. The barriers (identified in Sect. 3.2.3) are positioned according to the phase of technological transformation. Part of the barriers are positioned as antecedents (before technology introduction), and some barriers are positioned in the process of technological transformation (after introducing the technology). Enablers (presented in Sect. 3.2.4) are positioned in the process of technological transformation as they are considered its facilitators. The overall transformation is influenced by control conditions (presented in Sect. 3.2.5). Finally, the technological transformation process may result in three outcomes (as discussed in Sect. 3.2.8): level of technology assimilation, level of BMI, and performance outcomes.

## Technological transformation in SMEs: future research directions

Despite the significant number of papers identified on technological transformation in SMEs, the literature is still in development. We identify five research avenues that require further investigation, which are detailed as follows:

### Research avenue 1: Process of technological transformation

Scholars highlight the importance of concept/construct clarity and the mechanisms that link different constructs (Foss and Saebi 2017). In this regard, although previous studies suggest the overlap between technology assimilation and BMI processes, this interaction is not often addressed (Sabatini et al. 2022). While we provide a framework for technological transformation, it can be extended by improving the understanding of the process of technological transformation.

Firstly, future studies can contribute by providing insights into the integration of technology assimilation and BMI processes. To build a more integrative framework, future studies can rely on the connection between technology-related theories and BMI theories, which is still underexplored. Another possibility is to combine these theories with other complementary theories (e.g. resource-based view, dynamic capabilities, etc.).

Secondly, the phases of the technological transformation process remain abstract. The literature is not homogeneous in presenting phases for BMI (Wirtz and Daiser 2018), nor for the phases for technology assimilation (e.g. Fichman and Keremer 1997; Zhu et al. 2006), let alone technological transformation phases. From a practical perspective, SMEs will need to receive insights to improve their understanding of technological transformation as well as information regarding tools and best practices to make the transformation process smoother. Future studies can provide theoretical contributions by describing phases, tools and methods for the technological transformation process (including technology assimilation and BMI) in the context of SMEs. Finally, it is unclear how managers/owners deal with both processes simultaneously when introducing technology, and empirical studies (especially longitudinal studies) can provide more detailed information.

### Research avenue 2: Technological transformation in different settings and from different perspectives

There is a concentration of papers on specific technologies, mainly digital and 4.0 technologies, which have received increased attention due to new technological trends and the COVID-19 crisis. However, as the technology type conditions technological transformation, the studies' findings may not be extended to other technological contexts. Future research can dedicate efforts to different technological contexts and provide insight into (i) technological transformation paths for SMEs or (ii) BM patterns or configurations. For example, agri-food SMEs face pressure to comply with new trends in the food industry (e.g. waste management, extended shelf life, healthier food), which can be addressed by introducing food processing technologies (Rodgers 2016). In this regard, studies could provide paths (including a sequence of phases and strategies to overcome expected barriers of technological transformation within an organizational context) or BM configurations for these SMEs. The food processing technology is one example of a technological trend within a specific sector, but there are also other technological trends that future studies can address.

The technology transformation studies were mainly conducted in developed countries, which are considered years ahead of their counterparts in terms of technological transformation (Akpan et al. 2022). Thus, further research can analyze how technological transformation occurs in developing countries, providing insights into the specificities of this context. Finally, the studies identified mainly take the perspective of owner-managers of SMEs; future studies can consider the perspective of employees, which generally are the technology users. Taking the employees' perspective can provide insight into how users can better adapt to technological transformation.

### Research avenue 3: Outcomes of technological transformation

Like Foss and Saebi (2017) reported in their literature analysis, part of the motivation to conduct most of the identified studies is the potential benefits of technological transformation, such as reducing cost, optimizing processes, accessing markets, introducing new products, and ultimately improving financial performance. However, most of the benefits expected from technologies are mainly reported at the theoretical level by the studies; few studies report performance gains with technological transformation or evaluate the performance consequences of technological transformation. In this regard, qualitative and quantitative research can be conducted to evaluate technological transformation's performance outcomes. Studies can also define performance measures for different technologies according to their objectives and phase of transformation. Finally, as studies generally report low transformation levels (in terms of the level of technological assimilation and level of BMI), further research can analyze in which conditions SMEs achieve higher transformation levels.

### Research avenue 4: Risks and their mitigation in technological transformation in SMEs

Although the literature states that managing technological transformation in SMEs is challenging (Petzolt et al. 2022) and entails risks of failure (Nguyen et al. 2015), studies do not provide insights regarding SMEs that have experienced severe problems with technological transformation. Future studies can investigate cases where there is a discontinuation of technology use or severe consequences due to technological transformation, which can provide insights into the causes of failure and how to manage the risks of technological transformation. Moreover, future studies can also address the role and development of risk management tools for technological transformation in SMEs.

### Research avenue 5: BMI as a precursor of technology assimilation

Generally, the relationship discussed in the literature is technology assimilation that drives BMI (Sabatini et al. 2022), and little is known about how BMI can enable technology assimilation/adoption. In this regard, as BM may be seen as a supporting mechanism for technology adoption and scaling (Groot et al. 2019), future studies can evaluate how BMI can support SMEs in technological transformation. Future studies can assess how to build collaborative BMs or how technology providers may improve their value propositions to meet SMEs' needs, especially in the case of new and advanced technologies that are less accessible to SMEs (Akpan et al. 2022).

## Conclusion

SMEs face pressure to introduce new technologies due to different requirements and changes in the business setting. Studies recognize the importance of technology assimilation, BMI, and their interplay in achieving the outcomes expected from new technology. However, the current body of knowledge on technological transformation is fragmented, and previous studies do not explicitly cover these concepts. The dispersed body of knowledge provides a vague understanding of the technological transformation phenomenon in SMEs.

This work addresses this gap by performing a comprehensive SLR based on 165 peer-reviewed articles in which technology assimilation and BMI are presented within the context of introducing technology in SMEs. This SLR synthesize the current state-of-the-art research on technological transformation in SMEs and contributes by (i) presenting the evolution of the field, the primary study approaches, and technological trends; (ii) classifying the paper's foci within the field; (iii) integrating and positioning technological transformation factors; (iv) inferring the interrelatedness between technology assimilation and BMI; and (iv) providing a framework of technological transformation.

The findings indicate that technological transformation is a relatively new topic in the literature, and its combination remains abstract. The research domain is also concentrated in various technological contexts (e.g. digital technologies, e-commerce, and industry 4.0). In this sense, we develop a general framework for the subject, as the antecedents, processes, and outcomes were extracted from studies in various contexts.

The findings demonstrate that most of the papers focus on factors. These factors were identified, integrated, and positioned in the technological transformation framework. The analysis of factors and processes of technological transformation suggests an overlap between technology assimilation and BMI, and we provide insight regarding their relationships (three relationships were identified). Results also show a limited number of studies assessing the outcomes of technological transformation in SMEs, and that, generally, SMEs present a low transformation level.

Finally, the limitations in the keywords used to perform the paper's search impact this work. We used generic terms without considering keywords for specific technologies (e.g. industry 4.0, digitalization) since the scope of this paper is general. Secondly, we only selected papers that present the term “business model” since we were interested in papers that explicitly address the BMI concept. Thirdly, papers focusing on the individual streams (technology assimilation or BMI) were not selected since they are out of the scope. The authors’ subjectivity also limits the papers' analysis and classification because only the authors' viewpoints were contemplated.

## Data Availability

The datasets generated during and/or analysed during the current study are available from the corresponding author on reasonable request.

## Footnotes

1 We use the term “technology assimilation” to cover different terms used in the studies (e.g. technological innovation, technology adoption), given the literature fragmentation. The term was chosen due to its comprehensiveness and for consistency purposes.

2 We use the term “technological transformation” analogous to the term “digital transformation” used in business studies to refer to a comprehensive transformation in the organization due to new technology (e.g. Warner and Wäger 2019; Singh et al. 2020).

3 We considered the predominant objective/outcomes of the paper, but there are papers that present also results related to other foci (e.g. paper focused on the process that also describe factors).
